# Exploring the Evolutionary Characteristics of Food Security in China and the United States from a Multidimensional Perspective

**DOI:** 10.3390/foods13142272

**Published:** 2024-07-18

**Authors:** Chang Tang, Xiaoliang Xie, Guo Wei, Linglong Pan, Zihan Qi

**Affiliations:** 1 School of Science, Hunan University of Technology and Business, Changsha 410205, China; chnxxlp6688@hutb.edu.cn (X.X.);; 2 Changsha Social Laboratory of Artificial Intelligence, Changsha 410205, China; 3 Department of Mathematics and Computer Science, University of North Carolina at Pembroke, Pembroke, NC 28372, USA; guo.wei@uncp.edu; 4 School of Statistics and Mathematics, Shandong University of Finance and Economics, Jinan 250014, China

**Keywords:** food security, China–US, CRITIC–MEREC–MARCOS model, sustainable development

## Abstract

Against the backdrop of global warming, intensifying regional conflicts, deglobalization, and the spread of diseases, global food security is facing severe challenges. Studying the food security situation in China and the United States in depth can provide practical experience for formulating food security policies for countries around the world and promoting global food security governance. On the basis of a meticulous review of the evolving connotations of food security, this study adopts six dimensions—quantity security, quality security, circulation security, economic security, ecological resource security, and policy security—as breakthrough points to construct a framework consisting of food security evaluation indicator system comprising 29 specific indicators. The CRITIC–MEREC–MARCOS model is applied to evaluate the status of food security in China and the United States from 2000 to 2022, while the obstacle degree model (ODM) model is utilized to identify factors impeding food security between the two countries. The results indicate that the level of food security in China has shown slight fluctuations initially, followed by a steady upward trend. The gap with the United States is continuously narrowing. However, significant differences between China and the United States still exist in terms of economic security, ecological resource security, and policy security. Furthermore, due to the limited productivity of agricultural labor, scarcity of water and soil resources, and low efficiency in the use of fertilizers and pesticides, China’s food security is subject to economic and environmental constraints. The restrictions imposed by economic security and ecological resource security on China’s food security are showing an increasing trend year by year. For the United States, with the obstruction of grain exports and the increasing frequency of drought disasters, the impact of circulation security and ecological resource security on food security is becoming increasingly prominent. In the future, China and the United States should join hands to address challenges, actively promote international cooperation in food security, and drive sustainable development for humanity.

## 1. Introduction

Food is the lifeblood of a nation and the source of life for its people. Food security is the cornerstone of a nation’s prosperity and stability, holding significant strategic importance for national security and sustainable development. The world is currently facing an unprecedented era of profound transformation, characterized by a complex risk landscape arising from multiple challenges such as climate change, regional conflicts, deglobalization, and the spread of diseases. The external political, economic, and natural environments are rapidly evolving, posing severe tests and challenges to global food security [1,2]. According to the ‘The State of Food Security and Nutrition in the World (SOFI) 2023 released by the FAO, it is highlighted that an estimated 691 to 783 million people worldwide faced hunger in 2022, with 2.4 billion individuals experiencing moderate to severe food insecurity, accounting for approximately 29.6% of the global population [3]. Notably, the regions of South Asia and Sub-Saharan Africa, despite making significant progress from 2000 to 2015, continue to exhibit the highest levels of hunger globally. In 2023, their GHI score stood at 27.0, significantly surpassing that of Latin America, Southeast Asia, Western Asia, and North Africa [4]. Achieving the specific sustainable development goal of ‘Zero Hunger’ by 2030 poses a formidable challenge. Examining the Global Food Security Index (GFSI) reports reveals a continual deterioration in the global food environment, with significant risk events such as the 2019 coronavirus pandemic and the Ukraine conflict continually impacting the already fragile global food system. Following its peak in 2019, the GFSI began to exhibit a downward trend. Disparities in food security levels among nations further widened, accentuating the increasing inequalities within the global food system [5]. Moreover, with the increasing demand for animal-source foods and bioenergy in human society, there has been intense competition for limited resources such as land, water, labor, and capital in the production of food, feed, and fuel in recent years [6]. By 2050, the global demand for animal products is projected to increase by 60% to 70%, further exacerbating the need for feed, particularly in developing countries [7]. Taking China as an example, shifting dietary habits from traditional grain foods to animal-source foods has led to a rapid increase in demand for feed grains. In 2021, approximately 71% of China’s corn, 58% of oats, 34% of sorghum, and 24% of wheat were used for animal feed purposes [8]. Additionally, the production of biofuels also consumes significant agricultural crop resources. In 2022, approximately 37.8% of corn and 46.3% of soybean oil in the United States were used for biofuel production [9]. The intensifying competition for resources among food, feed, and fuel production in the future will directly lead to reduced food supply and increased food prices, thus affecting food security.

In this context, ensuring food security for a nation is of paramount importance. The task of ‘firmly holding the people’s rice bowl in one’s own hands’ is crucial. When it comes to global food security and sustainable development, the food security situation of China and the United States, as the two major nations, is undoubtedly a highly anticipated focus. As significant contributors to the global agricultural network and essential participants in the global agricultural system, China and the United States play crucial roles in safeguarding global food security. Moreover, the agricultural sector structures of both countries are quite similar, and both face many similar agricultural challenges, such as agricultural water supply issues, declining labor force, and food safety concerns related to genetically modified foods [10]. In-depth research into the food security situations of China and the United States contributes to advancing global food security governance and promoting human sustainable development.

Consequently, this study embarks from multiple dimensions including quantity, quality, circulation, economic, ecological resource, and policy. It employs the CRITIC–MEREC–MARCOS model to evaluate the food security conditions in China and the United States, and utilizes the obstacle degree model (ODM) to identify the factors impeding food security in both countries. The aim of this research is to elucidate the current issues and challenges confronting food security in China and the United States through a comparative analysis, thereby offering insights to inform the formulation and implementation of policies pertaining to food security strategies at the bilateral and global levels.

## 2. Literature Review

Food security is a complex issue, and understanding and grasping the essence of food security is the foundation for analyzing the situation of national food security. From the perspective of dialectical materialism and historical materialism, the concept of food security is relative, dynamic, and changing. Over the past few decades, the definition of food security by the United Nations Food and Agriculture Organization (FAO) has been continuously revised. Among them, the concept that ‘when all people, at all times, have physical, social and economic access to sufficient, safe and nutritious food that meets their dietary needs and food preferences for an active and healthy life’. is the most widely known [11]. Subsequently, food security is broadly understood as four key pillars, namely, availability, access, utilization, and stability. However, with the deepening research on food security issues, some studies have pointed out that the current four-pillar framework of food security can no longer comprehensively measure a country’s level of food security. It lacks analysis from the perspectives of sustainable agricultural production and policy, and the framework is conceptualized with specific indicators that are difficult to quantify [12,13]. Thus, how to rationally formulate a food security framework and measure the degree of food insecurity remains a highly challenging task.

Current research on food security measurement primarily focuses on two fronts: micro-level household surveys and macro-level multi-indicator comprehensive evaluations. A household survey refers to a sampling survey conducted to assess the food security status of regional households or individuals. The Food Consumption Score (FCS) is a composite score developed by the United Nations World Food Programme (WFP) based on a household’s dietary diversity, food consumption frequency, and the relative nutritional value of different food groups within a 7-day period to reflect the level of household food security [14]. The Household Food Security Survey Module (HFSSM) was developed by the U.S. government, and it assesses household food security status by asking 18 questions about the perceptions of food insecurity [15]. The LSMS-ISA, developed by the World Bank, is designed and implemented with a focus on agriculture-oriented household surveys, aiming to assess the food security of households and individuals in Sub-Saharan Africa [16]. Reichenheim (2016) proposed the integration of the Latent Class Factor Analysis (LCFA) model with the Brazilian Food Insecurity Measurement Scale (EBIA) for the classification of food insecurity in Brazilian households [17]. Maitra (2017) proposed a food security scale (KHFSSM) based on nine items to measure the food security of families in the slums of Kolkata, India [18]. There are also tools such as the Food Insecurity Experience Scale (FIES) [19], the Household Food Insecurity Access Scale (HFIAS) [20], and the Coping Strategy Index (CSI) [21]. In terms of the subject of this study, the United States has made substantial achievements in research on household food security surveys, but China still lacks research in this area [22,23,24]. Household surveys, from a micro perspective, can more intuitively reflect the state of household food security. However, the survey questions are somewhat subjective, and the actual implementation is relatively cumbersome, which is not conducive to evaluating the overall situation of a country.

A multi-indicator comprehensive evaluation is currently the most widely researched method. Starting from multiple dimensions, it can interpret the connotation of food security in more detail and better analyze the changes in the level of food security. The 2022 Global Food Security Index (GFSI), released by the EIU, takes into account the affordability, availability, quality, and safety of food, as well as sustainability and adaptability, and from this perspective, it quantifies 68 indicators to conduct an evaluation of the food security status of 113 countries worldwide [5]. Jennifer (2022) pointed out that the definition of food security needs to be updated and proposes a food security evaluation system based on six dimensions: availability, access, utilization, stability, agency, and sustainability [12]. Schindler (2018) proposed a method based on the Framework for Participatory Impact Assessment (FoPIA), conducting a food evaluation of rural Tanzania from three dimensions: social, economic, and environmental [25]. Wineman (2016) quantified indicators from three dimensions: quantity, quality, and stability, and utilized principal component analysis to conduct a multidimensional evaluation of food security in rural Zambia [26]. In terms of the subject of this study, China has a wealth of research on macro-level food security measurements. Jiang et al. (2023), based on the novel conceptualization of food security in the ‘Great Food View’, evaluated China’s provincial food security under the ‘Great Food View’ in three directions: quantity goal, quality goal, and stability goal [24]. Fei (2023) constructed a food security risk assessment model based on food production capacity, accessibility, availability, and stability, and analyzed the spatiotemporal changes in China’s food security pattern from micro-, meso-, and macro scales [27]. Wu (2016) embarked from the perspective of production–consumption coordination and matching, and based on the three dimensions of quantity coordination, structural coordination, and regional coordination, conducted an evaluation of China’s food security level [28]. Similar studies have also evaluated China’s food security from the perspectives of sustainable development [29,30] and synergistic development [31,32]. Given that research on food security measurements in China tends to lean towards macro-level multi-indicator evaluations while U.S. research tends to focus on micro-level household surveys, there is currently a lack of comparative studies on food security between China and the United States from a macro perspective.

According to the existing research literature, the academic community has conducted diverse studies on food security with rich results, which objectively reflect the development status of food security and lay a foundation for this study. However, there are several challenges in conducting further research on food security in China and the United States. Firstly, the indicator system is not unified and perfect. Variations exist in the research of food security indicator systems among different countries. Additionally, the comparative studies of agriculture between China and the United States are predominantly confined to policy-level analyses, with a conspicuous absence of systematic and comprehensive quantitative comparisons. A relatively comprehensive food security indicator system, applicable to both China and the United States, is yet to be established. Secondly, the food security evaluation methods require further refinement. Previous studies have typically employed methods such as the analytic hierarchy process (AHP) [33], entropy weight method (EWM) [24], principal component analysis (PCA) [26], TOPSIS [29], and matter element extension model (MEEM) [34]. In terms of index weighting, the AHP is too subjective to objectively reflect the importance of indicators. Although EWM and PCA are objectively weighted, PCA has low index interpretability, and EWM does not consider the correlation between indicators and is sensitive to data fluctuations, which can easily lead to unreasonable weight allocation. The Critic method, which considers indicator variation and correlation when constructing weights, effectively addresses the issue of overlooking indicator correlation in previous studies [35]. The MEREC method assigns weights by the removal effects of criteria, thereby better reflecting the impact of individual indicator changes on the overall evaluation [36]. The combination of the Critic and MEREC methods provides a more objective reflection of indicator weights. In terms of ranking evaluation methods, simple weighting cannot reflect real changes. The MEEM has a certain subjectivity and arbitrariness when determining the classical domain, which can easily lead to unstable results. Although the TOPSIS method is simple and effective, it does not consider the relative importance of each scheme and the positive and negative ideal solutions [37]. The MARCOS method, on the other hand, effectively overcomes the shortcomings of the previous evaluation methods, and its evaluation results are objective, stable, and reliable [38]. While the MARCOS method is a novel multi-criteria decision-making approach that has been applied in areas such as traffic risk assessment [39], human resources evaluation [40], and wind turbine site selection [41], it has not been applied in the field of food security evaluation until now.

Unlike previous research, this study is dedicated to a more targeted and representative comparative analysis of food security in both China and the United States. The primary contributions of this study can be summarized in three key aspects. First, this study has established a relatively comprehensive, systematic, and feasible evaluation system for food security in China and the United States. The evaluation framework focuses on six crucial aspects: quantity security, quality security, circulation security, economic security, ecological resource security, and policy security. Second, the introduction of the Critic–MEREC–MARCOS model offers a novel approach to evaluating food security, complemented by the utilization of the ODM to analyze the factors that pose obstacles to food security. Third, by conducting a systematic and qualitative comparative study between China and the United States, this research reveals the challenges and issues faced by both countries in terms of food security.

The structure of the remainder of this paper is as follows: Section 3 constructs the food security evaluation index system, detailing the reasons for the selection of each dimension and each index. Section 4 is about data and methods, providing a detailed introduction to the sources of data and the principles of the core model. Section 5 is an empirical study, elucidating the evolution of food security in China and the United States, and analyzing it in conjunction with national policies. Section 6 discusses the results, provides the final conclusions, and points out the deficiencies of the research.

## 3. Construction of Food Security System

### 3.1. Principles, Ideas, and Framework for Indicator System

In the process of designing the evaluation framework for food security, this paper comprehensively grasps the connotation of food security as defined by the Food and Agriculture Organization (FAO) of the United Nations, and strictly adheres to the principles of science, systematicity, relative independence, and data availability. Building upon previous research, this paper takes into account the macro-control role of national government agencies in the process of food production, as well as the requirements for sustainable agricultural production, and is subdivided into 29 quantifiable indicators from six dimensions: quantity security, quality security, circulation security, economic security, ecological resource security, and policy security. According to the China National Bureau of Statistics, this study primarily focuses on three main categories of food: grains, beans, and tubers. However, due to data collection constraints, grains are limited to wheat, corn, rice, oats, sorghum, and barley. Beans include only soybeans, and tubers encompass potatoes and sweet potatoes.

### 3.2. Composition of the Indicator System

(1) Quantity Security

The emphasis of food quantity security lies in increasing food production to ensure the capacity for supplying an adequate quantity of food, meeting the basic survival needs of the people, that is, to solve the problem of ‘Having enough to eat’. The issue of food quantity security is a primary concern for all countries, especially for developing nations such as those in Africa that are currently facing hunger. To ensure quantity security is to guarantee the effective supply of staple grains, ensuring stable and efficient food production that not only meets the macro-level total requirements of the nation but also safeguards the micro-level consumption needs of individuals. Thus, this study employs grain production volatility as a measure of the stability of food production and uses grain yield per unit area to assess production efficiency [29]. Additionally, total grain production is selected to reflect the scale of food production, while per capita grain possession reflects the supply capacity of food production under changing population dynamics [42].

(2) Quality Security

Quality security is proposed based on the societal function of improving living standards through food consumption. With the progression of time, the provision of food necessitates ensuring not only quantity but also quality in supply. The quality security of food has emerged as an elevated requirement for food security in the new era. Ensuring quality security is to better meet people’s higher demands for the nutritional, healthful, and diverse aspects of food; conform to the trend in food structure changes; and realize the transition from ‘eating enough’ to ‘eating healthily and safely’ [43]. In general, the use of indicators such as bulk density, moisture content, impurities, and milling yield can directly measure the grain quality [44]. However, considering the level of data disclosure in different countries, these indicators are not readily available. Furthermore, due to the substantial number of missing data points for indicators such as the prevalence of undernourishment, micronutrients (iron, zinc, and vitamins) and their inclusion in the assessment was also omitted to avoid potential bias. Therefore, this paper selects internationally recognized nutrients (proteins, fats, and dietary energy) to measure the nutritional and health levels of food acquisition, and the share of dietary energy supply from cereals, roots, and tubers to measure the diversity level of food acquisition [5,45].

(3) Circulation Security

Food circulation acts as a bridge between food production and consumption, covering aspects like storage, transportation, sales, and trade. Ensuring the smooth supply of grains from producers to consumers is an indispensable segment in the grain supply chain. Circulation security focuses on addressing the issue of ‘available’. Food transportation is a crucial component of food circulation, and the current modes of grain transportation in China–US food logistics primarily rely on railways, roadways, and waterways [46]. Considering the difficulties in obtaining data on waterway transportation and food loss, this study selects the density of railway and highway transportation routes and their freight turnover indicators to indirectly measure the basic transportation capacity of food [34]. In terms of food storage and sales, this paper reflects on the grain reserve situation, and the emergency dispatching ability of grain during grain production is obstructed by calculating the stock to use ratio [29]. Additionally, the domestic per capita grain consumption is calculated to measure the food sales situation. The globalization of food trade effectively links the food security of various countries. The grain self-sufficiency ratio is calculated to reflect the degree of external dependence on grain, indirectly indicating the state of grain trade.

(4) Economic Security

Food economic security starts from the perspective of food acquisition, examining people’s economic ability to acquire food, and focuses on solving the problem of ‘affordable’. Economic security is primarily measured from the three perspectives of purchasing power, production efficiency, and price stability. The purchasing power of residents for food is primarily influenced by personal income, consumption structure, and social welfare. Therefore, indicators such as per capita disposable income, Engel’s coefficient of residents, and poverty incidence rate are selected to reflect the purchasing power of residents for food. Agricultural labor productivity depicts the efficiency and benefits of agricultural production, and is a crucial gauge of the level of agricultural economic and technological development. Excessive volatility in grain prices can significantly disturb the equilibrium of food supply and demand, influencing the production enthusiasm of farmers on the supply side and the purchasing desire of consumers on the demand side. Maintaining the operation of grain prices within a reasonable range is conducive to promoting the sustainable development of the economic aspects of food production. Thus, the food price consumer index is selected to reflect the fluctuations in food prices [33].

(5) Ecological Resource Security

Ecological resource security emphasizes the examination of the sustainability of food production under resource constraints. The agricultural ecological environment, as the carrier of food production, has a significant impact on both the yield and quality of crops [32]. Balancing the relationship between ‘production’ and ‘ecology’ is the key to sustainable production. Measuring the ecological resource security level is performed by selecting specific indicators from the aspects of natural resource supply, production factor input, and climate change. For natural resource supply, two specific indicators were selected: water resources and arable land. Regarding production input elements, three indicators were selected, namely, the amount of pesticides and fertilizers used per unit, and the effective irrigated area. Pesticides and fertilizers are significant contributors to the degradation of water quality, soil contamination, and the overall deterioration of the agricultural ecosystem. Their excessive use poses a serious threat to the sustainable development of food production [47]. The area of irrigated arable land measures the sustainability of food production from the perspectives of water resource utilization efficiency and usage scale. As the trend in global warming progressively intensifies, the implications of extreme climatic events, induced by climate change, on food security have elicited extensive concern across various societal sectors. Drought, being one of the principal calamities confronting the agricultural system, significantly jeopardizes global food security and the sustainable progression of the agricultural system [48]. Given the accessibility of data, this study solely adopts the proportion of drought-affected areas as a specific indicator to measure the security of ecological resources.

(6) Policy Security

Policy security focuses on the macro-control role of the national government in ensuring food security. Research has shown that an aggregate governance metric can effectively assess food security [49]. Political stability, which measures the stability of the regime in the country, is positively correlated with food security [45]. Political stability is a comprehensive manifestation of social order, the rule of law environment, and policy continuity and implementation, and has a fundamental and safeguarding role in food policy security. Government effectiveness, or effective government management, can fully leverage the dominant position of the government and the regulatory function of the market, ensuring that the promulgation of food policies is scientific, targeted, and effective. Regulatory quality is essential. Effective regulation not only ensures the quality and safety of agricultural products, thereby safeguarding consumer health and rights, but also provides timely insights into market supply and demand dynamics, optimizing the production structure of the food industry. Agricultural research funding expenditure is pivotal for enhancing agricultural productivity, quality, and competitiveness. It also serves as a significant pillar for strategies aimed at promoting green agricultural development, the construction of digital agriculture, and the revitalization of the seed industry, exerting a profound influence on the security of food policy. The specific indicators are shown in Table 1.

## 4. Methods and Data

### 4.1. Data Sources

This study conducts a comprehensive comparative analysis of the food security situation in China and the United States from 2000 to 2022. The research data mainly come from the National Bureau of Statistics, the Ministry of Transport of China, *the China Rural Statistical Yearbook, the China Grain and Materials Reserve Yearbook, the China Statistical Yearbook, the China Statistical Yearbook On Science and Technology*, the Food and Agriculture Organization of the United Nations (FAO), the World Bank, the U.S. Bureau of Economic Analysis, the U.S. Department of Labor, the U.S. Department of Transportation, the U.S. Department of Agriculture, the U.S. National Integrated Drought Information System, as well as Bric Agricultural Data Intelligence Terminal, and STATISTA Database. Some indicators are calculated based on the existing literature, statistical bulletins, and official government websites. For years with missing data, curve fitting is employed to approximate estimates, thereby maintaining data integrity. The specific sources are shown in Table 2.

### 4.2. Research Method Selection

#### 4.2.1. Weighting the CRITIC Method

The CRITIC method, based on the intensity and conflict of inter-indicators, overcomes the shortcomings of the entropy method that cannot take into account the correlation of indicators, and can more objectively reflect the weight of indicators [35]. Contrary to prior research, the study employs the coefficient of variation instead of the standard deviation to measure the degree of data variability. At the same time, the degree of conflict of indicators should be independent of the positive or negative degree of indicator correlation. Therefore, we take the absolute value of the correlation coefficient to eliminate the impact of its sign. The following are the steps to calculate the objective weights using the CRITIC method.

Step 1: Normalize the processing of data. Suppose the original decision matrix is X, where $x_{ij}$ represents the specific value of the *i*-th evaluation object and the *j*-th evaluation indicator. There are a total of m evaluation objects and n evaluation indicators.

(1) $$X=\left[\begin{array}{cccc} x_{11} & x_{12} & \cdots & x_{1n}\\ x_{21} & x_{22} & \cdots & x_{2n}\\ \vdots & \vdots & \ddots & \vdots \\ x_{m1} & x_{m2} & \cdots & x_{mn} \end{array}\right]$$

Due to the different units and dimensions of each indicator, it can affect the evaluation. Therefore, we perform standardization processing on each indicator variable. Positive indicators apply to Formula (2), while negative indicators apply to Formula (3).

(2) $$x^{\prime }{\;\;}_{ij}=\frac{x_{ij}-\min(x_{ij})}{\max(x_{ij})-\max(x_{ij})}$$

(3) $$x^{\prime }{\;\;}_{ij}=\frac{\max(x_{ij})-x_{ij}}{\max(x_{ij})-\max(x_{ij})}$$

In accordance with standardization, a standardized matrix $X^{\prime }=[x_{ij}^{\prime }]$ was obtained.

Step 2: Determine the objective weights. $c_{i}$ denotes the information content, ${CV}_{i}$ represents the coefficient of variation of the j-th indicator, $r_{kj}$ is the correlation coefficient between the *j*-th and *k*-th indicators, and $w_{j}^{\prime }\;$ is the weight assigned to the *j*-th indicator.

(4) $$c_{j}=CV_{j}\sum_{k=1}^{m}\left(1-|r_{kj}|\right)$$

(5) $$w_{j}^{\prime }=c_{j}/\sum_{j=1}^{n}c_{j}$$

#### 4.2.2. Weighting the MEREC Method

MEREC (method based on removal effects of criteria), proposed by Mehdi in 2021 [36], is a novel objective weighting method designed to address the issue of indicator weights in multi-criteria decision making (MCDM). Unlike traditional objective weighting methods, which are largely based on changes in indicators, MEREC determines the weights of each indicator from the perspective of indicator elimination. If the elimination of a certain indicator has a significant impact on the overall evaluation result deviation, then this indicator will be assigned a greater weight. The specific calculation steps are as follows.

Step 1: Normalize the decision matrix *X*. Use the minimization-type standardization to scale the elements of the decision matrix. The formula is as follows:

(6) $${x^{\prime }}_{ij}=\left\{\begin{array}{c} \frac{\min_{k}x_{ij}}{x_{ij}},\;if\;j\in Positive\;indicators\\ \frac{x_{ij}}{\max_{k}x_{kj}},if\;j\in negative\;indicators \end{array}\right.$$

Step 2: Calculate the overall performance $Q_{i}$ of the alternative solutions under the complete set of indicators. Use the measure function to evaluate the alternative solutions, where the measure function used is a non-linear logarithmic function, $f\left(x\right)=\ln\left(1+|\ln\left(x\right)|\right)$. Among them, $Q_{i}$ stands for the overall performance of the i-th alternative solution.

(7) $$Q_{i}=\ln\left(\left.1+\left(\left.\frac{1}{m}\sum_{j=1}^{m}\left|\ln(x_{ij}^{\prime })\right|\right)\right.\right)\right.\left(i=1,2,\cdots n;j=1,2,\cdots ,m\right)$$

Step 3: Calculate the overall performance $Q_{ij}^{\prime }$ of the alternative solutions after each indicator is removed. This step is consistent with step 2, still using a non-linear logarithmic function as the measure function. $Q_{ij}^{\prime }$ describes the overall performance of the *i*-th alternative solution after the *j*-th indicator is removed.

(8) $$Q_{ij}^{\prime }=\ln\left(\left.1+\left(\left.\frac{1}{m}\sum_{k,k\neq j}\left|\ln(x_{ik}^{\prime })\right|\right)\right.\right)\right.\left(i=1,2,\cdots n;j=1,2,\cdots ,m\right)$$

Step 4: Calculate the total sum of absolute deviations. The degree of the impact of the *j*-th indicator on the overall evaluation effect can be calculated from $Q_{i}$ and $Q_{ij}^{\prime }$ obtained from the previous two steps. Among them, $E_{j}$ represents the change in the overall evaluation result after the *j*-th indicator is removed, which is used to reflect the impact of the *j*-th indicator on the evaluation result.

(9) $$E_{j}=\sum_{i=1}^{n}\left|Q_{ij}^{\prime }-Q_{i}\right|;\left(i=1,2,\cdots n;j=1,2,\cdots ,m\right)$$

Step 5: Determine the final objective weights. Based on the effect of the indicator removal $E_{j}$, calculate the weights of each indicator. $w_{j}^{\prime \prime }\;$ represents the weight of the *j*-th indicator.

(10) $$w_{j}^{\prime \prime }=E_{j}/\sum_{j=1}^{m}E_{j}\;\;\;(j=1,2,\cdots ,m)$$

#### 4.2.3. Cooperative Game Theory-Based Weight Optimization

To mitigate the limitations of a single objective weighting method, this study employs game theory-based composite weighting to optimize the weight values obtained from various objective weighting methods mentioned above, resulting in a more rational weight allocation [50].

Step 1: Construct the combination weight vector. Assuming there are L weighting methods, where the basic weight vector for the *k*-th method is denoted as $w_{k}=\{w_{k1},w_{k2},\cdots w_{kn}\}(k=1,2\cdots ,L),$ combine the *L* weight vectors linearly to create the combination weight vector W. In the formula, $a_{i}$ represents the weight allocation coefficient.

(11) $$W=\sum_{i=1}^{l}a_{i}w_{i}^{T}$$

Step 2: Establish the objective function. Minimize the sum of deviations between the combination weights and the basic weights. Continuously optimize the weight allocation coefficients $a_{i}$ to minimize the deviation between *W* and ${\;w}_{i}$. The formula is given as follows:

(12) $$\min\sum_{K=1}^{L}||\sum_{i=1}^{l}a_{i}w_{i}^{T}-w_{k}^{T}|{|}_{2}$$

Step 3: Resolve the objective function to calculate the combination weights. By leveraging the differential properties inherent in matrices, the first-order derivative condition for Equation (12) can be ascertained.

(13) $$\sum_{i=1}^{L}a_{i}\cdot w_{k}\cdot w_{i}^{T}=w_{k}\cdot w_{i}^{T}$$

The corresponding linear system of equations is as follows:

(14) $$\left[\begin{array}{cccc} w_{1}\cdot w_{1}^{T} & w_{1}\cdot w_{2}^{T} & \cdots & w_{1}\cdot w_{L}^{T}\\ w_{2}\cdot w_{1}^{T} & w_{2}\cdot w_{2}^{T} & \cdots & w_{2}\cdot w_{L}^{T}\\ \vdots & \vdots & \ddots & \vdots \\ w_{L}\cdot w_{l}^{T} & w_{L}\cdot w_{2}^{T} & \cdots & w_{L}\cdot w_{L}^{T} \end{array}\right]\left[\begin{array}{c} a_{1}\\ a_{2}\\ \vdots \\ a_{L} \end{array}\right]=\left[\begin{array}{c} w_{1}\cdot w_{1}^{T}\\ w_{2}\cdot w_{2}^{T}\\ \vdots \\ w_{L}\cdot w_{L}^{T} \end{array}\right]$$

Subsequently, the optimal weight allocation coefficients $\left(a_{1,}a_{2,}\cdots a_{L}\right)$ are derived based on the aforementioned equation, followed by a process of normalization:

(15) $$a_{i}^{*}=\frac{a_{i}}{\sum_{i=1}^{L}a_{i}}$$

Hence, the final combination weight is determined as follows:

(16) $$w^{*}=\sum_{i=1}^{L}a_{i}^{*}w_{i}^{T}$$

#### 4.2.4. MARCOS Method

The MARCOS method is a novel multi-criteria decision-making method, first proposed by Stević in 2020 [38]. This method is based on the relationships between the ideal solution, the negative ideal solution, and the alternative schemes, and introduces the concept of a utility function. This function is used to compromise rank alternative schemes based on their effectiveness values. The effectiveness function is used to measure the relative position of an alternative scheme between the optimal ideal solution and the worst ideal solution. The optimal alternative scheme is closest to the ideal value reference point and furthest from the negative ideal reference point. In this paper, the ideal reference point represents the highest degree of food security, and the alternative schemes (evaluation objects) refer to the food security situation in China and the United States over the years. The specific calculation steps are as follows:

Step 1: Form the extended initial decision matrix. In this step, the ideal solution (*AI*) and the negative ideal solution (*AAI*) are defined and introduced into the initial decision matrix *X* to extend it.

(17) $$X_{ext}=\begin{array}{c} AAI\\ A_{1}\\ A_{2}\\ \vdots \\ A_{m}\\ AI \end{array}\left[\begin{array}{cccc} x_{aai1} & x_{aai2} & \cdots & x_{aaim}\\ x_{11} & x_{12} & \cdots & x_{1m}\\ x_{21} & x_{22} & \cdots & x_{2m}\\ \vdots & \vdots & \vdots & \vdots \\ x_{n1} & x_{n2} & \cdots & x_{nm}\\ x_{ai1} & x_{ai2} & \cdots & x_{aim} \end{array}\right]$$

The negative ideal solution (*AAI*) and the ideal solution (*AI*) indicate the alternative with the worst characteristics and the alternative with the best characteristics, respectively. Depending on the different attributes of the indicators, *AAI* and *AI* are defined as follows:

(18) $$AAI=\left\{\begin{array}{l} \underset{1\leq i\leq n}{\min}x_{ij},j\in B\\ \underset{1\leq i\leq n}{\max}x_{ij},j\in C \end{array}\right.$$

(19) $$AI=\left\{\begin{array}{l} \underset{1\leq i\leq n}{\max}x_{ij},j\in B\\ \underset{1\leq i\leq n}{\min}x_{ij},j\in C \end{array}\right.$$

In the above formula, *B* stands for the positive indicators, and *C* stands for the negative indicators.

Step 2: Normalize the extended initial matrix. The extended initial decision matrix $X_{ext}$ is transformed into the normalized matrix $N={{\;[n}_{ij}]}_{(n+2)m}\;$ through the normalization formula.

(20) $$n_{ij}=\left\{\begin{array}{l} \frac{x_{ai}}{x_{ij}},j\in C\\ \frac{x_{ij}}{x_{aai}},j\in B \end{array}\right.$$

In the above formula, $x_{ai}$ and $x_{aai}$ are elements from the extended initial matrix, and $n_{ij}$ illustrates the elements in the matrix after the extended initial matrix is normalized.

Step 3: Calculate the weighted matrix $V={\;[v_{ij}]}_{(n+2)m}$. By multiplying each element $n_{ij}$ in the normalized matrix by the corresponding weight $w_{j}$, the weighted matrix is obtained.

(21) $$v_{ij}=n_{ij}\times w_{j}$$

Step 4: Calculate the utility degree $K_{i}$. The utility degree of each alternative scheme relative to the ideal solution and the negative ideal solution is calculated using the formula below.

(22) $$K_{i}^{+}=\frac{S_{i}}{S_{AAI}}$$

(23) $$K_{i}^{-}=\frac{S_{i}}{S_{AI}}$$

In the above formula, $S_{i}$ represents the sum of the elements in the *i*-th row of the weighted matrix, $S_{AAI}$ denotes the sum of the elements in the *AAI* row of the weighted matrix, and $S_{AI}$ denotes the sum of the elements in the *AI* row.

Step 5: Determine the utility function. The value of the utility function is a compromise result of the alternative scheme relative to the ideal solution and the negative ideal solution. The larger the value of the utility function, the better the alternative scheme, with a value range of [0, 1]. The specific definition is as follows:

(24) $$f(K_{i})=\frac{K_{i}^{+}+K_{i}^{-}}{1+\frac{1-f(K_{i}^{+})}{f(K_{i}^{+})}+\frac{1-f(K_{i}^{-})}{f(K_{i}^{-})}}$$

$f(K_{i}^{+})$ and $f(K_{i}^{-})$ describe the effectiveness functions related to the ideal solution and the negative ideal solution, respectively. They are defined as follows:

(25) $$f(K_{i}^{-})=\frac{K_{i}^{+}}{K_{i}^{+}+K_{i}^{-}}$$

(26) $$f(K_{i}^{+})=\frac{K_{i}^{-}}{K_{i}^{+}+K_{i}^{-}}$$

#### 4.2.5. Obstacle Degree Model

The obstacle degree model is employed to identify the key variables that constrain the food security of China and the United States, thereby seeking a path for the sustainable development of food security in both countries [34].

(27) $$O_{j}=\frac{(1-x_{ij}^{\prime })\cdot w_{j}}{\sum_{j=1}^{n}\left[(1-x_{ij}^{\prime })\cdot w_{j}\right]}$$

In the above formula, where $x_{ij}^{’}$ represents the standardized value of an individual indicator, $w_{j}$ denotes the weight of the corresponding indicator, $O_{j}$ signifies the obstacle degree, and *j* is used to indicate the magnitude of the impact of the *j*-th indicator on food security.

## 5. Results

### 5.1. Determination of Weights of Indicators

**Figure 1:**
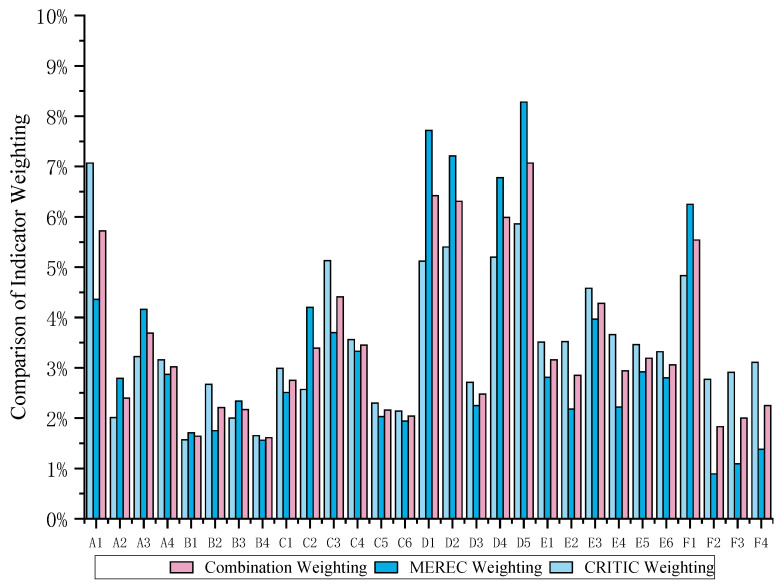
Comparison of indicator weighting.

By employing the Critic method and MEREC method, as previously illustrated, to assign weights to the food security indicators, we can derive the weights for the six dimensions and the weights of each indicator within these dimensions. The assignment of weights in the Critic method is computed in accordance with Equations (1)–(5), while the assignment of weights in the MEREC method is computed following Equations (6)–(10). The weighting differences between the MEREC method and the Critic method are assessed using the Pearson correlation coefficient. [36]. The Pearson correlation coefficient of the weights between the two methods is 0.7569, which is significantly correlated at the significance level of α = 0.01. This indicates that the two methods are essentially consistent in assigning weights to food security indicators. To mitigate the discrepancies in the assignment of weights to individual indices between the two methods, this study employs a combination weighting approach grounded in cooperative game theory [50]. The combination weights are computed utilizing Equations (11)–(16). Figure 1 compares the three methods for assigning weights to indicators. As shown in Figure 1, the combination weights are actually a compromise between the weight assignments by the MEREC and CRITIC methods.

As discerned from Table 3, within the various dimensions of food security, the weights for quantity security, quality security, circulation security, economic security, ecological resource security, and policy security are, respectively, 14.82%, 7.63%, 18.20%, 28.26%, 19.48%, and 11.61%.

Economic security holds the highest weight, followed by circulation security and ecological resource security, while quality security is relatively low. This shows that both China and the United States place a high emphasis on economic security, circulation security, and ecological resource security, while quality security is to some extent neglected. Upon the examination of specific indicators, it is evident that agricultural labor productivity (D5), per capita disposable income (D1), poverty incidence rate (D2), food price volatility (D4), and grain production volatility (A1) are crucial factors influencing food security in both China and the United States.

### 5.2. Comprehensive Evolution of Food Security Levels in China and the United States

**Figure 2:**
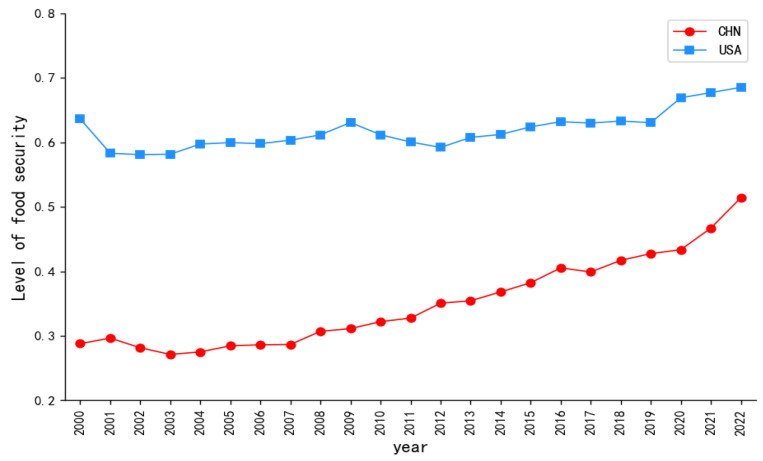
Changes in food security levels in China–US from 2000 to 2022.

Based on Formulas (17)–(27) in the MARCOS model, the overall scores of the food security levels in China and the United States over the past 23 years have been calculated. Figure 2 depicts the evolution of the overall development level of food security in China and the United States from 2000 to 2022. The level of food security in the United States has shown noticeable fluctuations during this period, but the overall level is relatively high and exhibits a slow upward trend. Contrastingly, China began with a lower baseline of food security compared to the United States, yet it developed at a faster pace. During this period, China’s food security level demonstrated a slight fluctuation, but the overall level continued to improve, and the gap in the food security levels between China and the United States was gradually narrowing. As shown in the latest 2022 GFSI report, over the past decade, China’s food security score has seen a net increase of 13.7 points, ranking second in growth rate among 113 countries, while the United States has only increased by 1.3 points, thus narrowing the ranking gap between China and the United States [5]. This indirectly validates the reasonableness of the results of this study.

**Figure 3:**
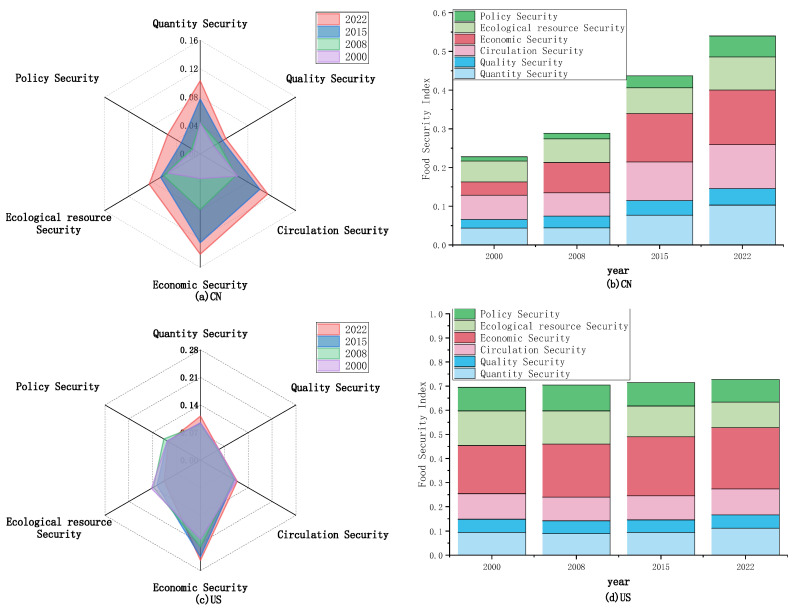
Results of food security evaluation. (**a**,**c**) Radar charts depict the changes in the six dimensions for CN and US across different years, and (**b**,**d**) histograms show the weights of each dimension for CN and US in different years.

Specifically, the level of food security in China can be delineated into three distinct phases: an initial decline, followed by a gradual ascent, and culminating in a rapid surge. The period from 2001 to 2003 marks a phase of decline, during which the level of food security in China fell from 0.2965 to 0.2712. In 2000, in order to adjust and optimize the structure of agriculture and food production, China issued the ‘*Notice of the State Council on Further Improving the Policy Measures for the Reform of the Food Circulation System*’ which abolished the protective price purchase range for spring wheat in the three northeastern provinces (Heilongjiang, Jilin, and Liaoning), eastern Inner Mongolia, northern Hebei, northern Shanxi; early indica rice in the south; and wheat in Jiangnan [51]. This meant that farmers could only plant according to market demand. Starting the same year, with the aim of alleviating fiscal pressure and compensating for the shortage of food production, China began to address a large amount of stored grain. As shown in Figure 6b, the stock to use ratio has been declining. Due to the government’s reduction in purchase prices and proactive de-stocking, grain prices have been depressed, leading to a decline in farmers’ enthusiasm for grain production. In 2001, with China’s entry into the WTO, contradictions in China’s economic operation began to emerge. Industries such as manufacturing and real estate experienced rapid investment growth, leading to a significant shift of land and capital towards non-agricultural sectors, thus exacerbating economic imbalances in China. These factors ultimately resulted in a severe decline in the sown area of grains and a substantial reduction in grain production, intensified fluctuations in grain yields, and marked decreases in both quantity and policy security. The period from 2004 to 2008 represents a phase of gradual ascent, with China’s food security level rising from 0.2751 to 0.3070. During this period, to ensure food security and increase farmers’ income, a series of policies supporting ‘agriculture, rural areas, and farmers’ and improving the comprehensive production capacity of agriculture were successively issued in the *China’s No.1 Central Document* released after 2004. The Chinese government has enacted a number of supportive policies, including the abolition of agricultural taxes, agricultural machinery subsidies, direct grain subsidies, quality seed subsidies, and the protection of the minimum purchase price for main grains. These measures have stimulated the enthusiasm of farmers and promoted agricultural production [52,53]. At the same time, reforms were made to the food circulation system, allowing the operators of diverse ownership to participate in the food market, thereby establishing a competitive food market [54]. The levels of economic security and circulation security in China have significantly improved. However, China’s extensive agricultural production model remained unchanged. For the purpose of increasing food production, the use of pesticides and fertilizers continued to rise, which led to a continuous decline in the level of ecological resource security. Therefore, during this stage, the overall increase in China’s food security level was not significant. The period from 2009 to 2022 marks a phase of rapid ascent, with China’s food security level soaring from 0.3113 to 0.5142. Throughout this timeframe, the Chinese government implemented a series of measures to safeguard food security, executed the strategy of sustainable farmland use and the innovative application of agricultural technology to increase farmland productivity. The implementation of the rural revitalization strategy and precise poverty alleviation policies have reduced poverty, developed the rural agricultural economy, and increased farmers’ income. The advancement of agricultural supply-side reform and institutional mechanism innovation ensure the high-quality and effective supply of agricultural products. China’s food production capacity is continuously strengthening, the level of modernization in food circulation has significantly improved, and the structure of food supply is constantly being optimized [55]. From the perspective of dynamic changes across the various dimensions in China, as illustrated in Figure 3a, China prioritized quantity security, circulation security, and ecological resource security in the initial stages. Gradually, China began to pay attention to economic security, policy security, and quality security, progressively moving towards a multidimensional development. However, an overarching observation is the persisting inadequacy in China’s emphasis on quality security and policy security. 

There exist two distinct periods of decline in the level of food security in the United States, specifically from 2000 to 2002 and from 2010 to 2012. During the period from 2000 to 2002, the food security level in the United States declined from 0.6364 to 0.5810. Towards the end of the 1990s, due to overproduction and economic recession in Asia and Latin America, agricultural prices began to plummet, ultimately leading to a significant reduction in U.S. exports [56]. Concurrently, the *Federal Agriculture Improvement and Reform Act* of 1996 led to the reduction or elimination of many subsidies and restrictions. Furthermore, domestic grain stocks in the U.S. were rapidly depleted, exerting upward pressure on prices. As shown in Figure 6b, the stock to use ratio consistently declined. Coupled with an oversupply that kept food prices low, the overall U.S. agricultural economy was in a slump, with sharp declines in farmers’ earnings and a decrease in production enthusiasm [57]. Compounding this, most regions in the U.S. faced persistent or worsening droughts, resulting in a severe lack of water resources for crops during the growth period [58,59]. This led to a reduction in sown area and a sharp decrease in grain production. During this period, the levels of quantity security, circulation security, and ecological resource security in U.S. food security experienced varying degrees of decline. During the period from 2009 to 2012, the level of food security in the United States declined from 0.6309 to 0.5922. During this period, the biofuel policies in the United States introduced a series of risks to food security. The implementation of U.S. biofuel incentive policies led to an expansion in the cultivation area of crops such as corn and soybeans. However, this was accompanied by an increase in marginal and unsuitable lands for cultivation, leading to a decline in the overall quality of arable land [60]^.^ Moreover, the increase in biofuel production is impacting the supply of food in the United States. From 2000 to 2010, the average share of corn used for biofuels increased from 6.47% to 38.51%, while corn production only grew by 5.24% [9]. Simultaneously, biofuel policies have led to an escalation in food prices [61], and the United States was in a period of recovery following an economic recession (2007–2009), resulting in a proportion of household food insecurity during this time that remained higher than before the economic downturn [23]. In terms of climate, the resurgence of the La Niña phenomenon has once again led to widespread drought in the United States, with the drought coverage rate increasing year by year, peaking in 2012 [62]. This ultimately led to a decline in U.S. grain production for three consecutive years. The levels of quantity security, ecological resource security, and economic security in the United States have all experienced certain declines. From the perspective of dynamic changes across the various dimensions in the United States, as illustrated in Figure 3c, notwithstanding significant alterations in ecological resource security and economic security, the magnitudes of change in circulation security, quantity security, and quality security have remained comparatively stable from 2000 to 2022. The United States has consistently accorded high priority to economic security, yet a discernible decline in attention towards ecological resource security is evident, and the emphasis on quality security remains insufficient.

### 5.3. Comparison of Changes in Various Dimensional Subsystems of Food Security

#### 5.3.1. Quantity Security

**Figure 4:**
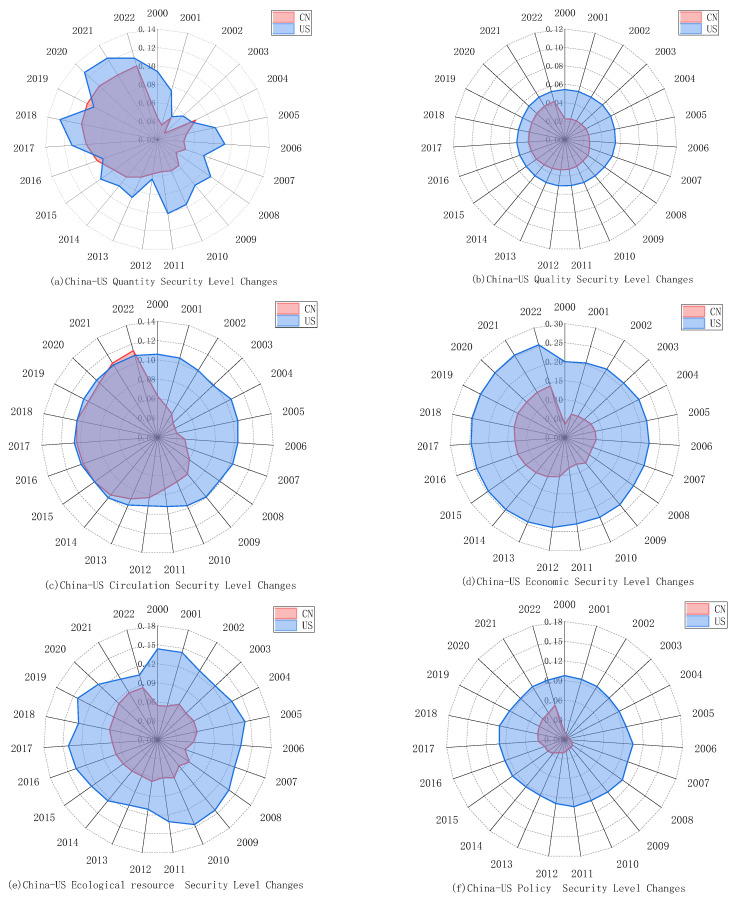
Trends in changes in food security subsystems in CN and US from 2000 to 2022.

As illustrated in Figure 4a, the level of quantity security in China experienced minor fluctuations prior to 2004, subsequently rising year by year. The level of food quantity security in China has risen from 0.0434 in 2000 to 0.1031 in 2022, indicating a significant increase in magnitude. In comparison, the overall level of food quantity security in the United States is slightly higher than that in China. However, with the rapid development of China, this disparity is steadily diminishing. Notably, the quantity security in the United States has exhibited significant fluctuations, likely influenced by factors such as extreme weather, animal diseases, or pest infestations, indicating potential risks in its stability. 

**Figure 5:**
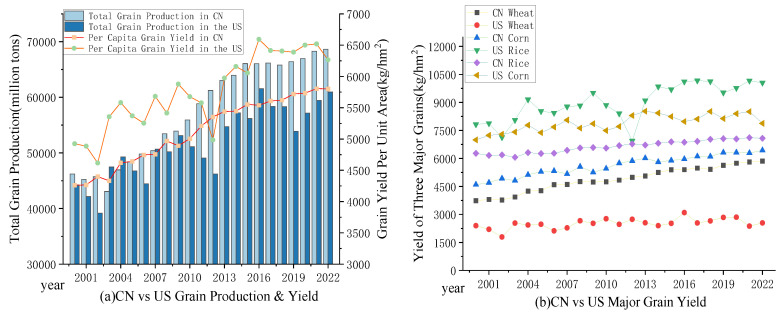
A comparative study of grain supply in China and the United States from 2000 to 2022; data source: the National Bureau of Statistics of China and the U.S. Department of Agriculture.

In terms of grain production, China achieved its nineteenth consecutive year of abundant grain harvest in 2022, with a significant decrease in the fluctuation rate of grain output and a noticeable increase in grain yield. The total grain output in China reached 686.528 million tons in 2022, the per unit yield of grain reached 5801.7 $kg/{hm}^{2}$, and per capita grain possession reached 486.29 kg, exceeding the United Nations standard of 400 kg [63]. The specific trend changes are shown in Figure 5a. With the continuous enhancement of China’s innovative capabilities and technological promotion for augmenting food production, the advancement of modern efficient agriculture and the level of large-scale food operation have collectively fortified the bedrock of food quantity security [64]. For instance, the groundbreaking work of Yuan Longping’s team in the development of third-generation hybrid rice and Saline-alkali tolerant rice; the innovative creation of the compact, large panicle hybrid corn variant ‘Denghai 618’ by Li Denghai’s team; and the pioneering efforts of Li Zhensheng’s team in the development of the distant hybridization wheat variety ‘Xiaoyan’ series. These scientific advancements have collectively propelled a positive trajectory in augmenting China’s grain yield. Relative to the United States, China manifests superior prowess in terms of aggregate grain production and the steadiness of grain yield (fluctuations in grain production). Yet, when it comes to the efficiency of grain production, China still has a gap to bridge with developed nations such as the United States. On average, China’s grain yield per unit area is only 88.45% of that in the United States, with the per unit area yields of corn and rice in the U.S., excluding wheat from the three main grains, being 1.61 and 1.19 times that of China, respectively, as specifically illustrated in Figure 5b. The reasons for this can be primarily attributed to two factors. First, there is a difference in production scale. Currently, China faces a shortage of per capita arable land resources, with severe land fragmentation and smallholder agriculture predominating, which significantly hampers the progress of large-scale operations and the modernization of agriculture, thereby limiting the improvement of agricultural production efficiency [65,66]. In contrast, since the 20th century, the scale of farm production in the U.S. has been continuously expanding [67]. By 2017, farms with more than 2000 acres of cropland accounted for 58% of the total cropland [68]. The vast and flat terrain facilitates the comprehensive implementation of mechanization and intelligent production. Second, there is a disparity in the level of agricultural production technology. Although China has made key breakthroughs in areas such as crop breeding, intelligent production, and pest and disease warning systems [69], limited government funding, inadequate grassroots management, and the low education level of extension personnel mean that only a small number of farmers benefit from public extension services [70]. Consequently, China’s innovation and information technology levels remain relatively low. Meanwhile, the U.S. has fully realized mechanized production, with crops such as corn and soybeans widely adopting high-yield genetically modified seeds, and is currently transitioning towards precision and intelligent agriculture [67,71,72]. Of course, there are differences in aspects such as subsidy policies and climate conditions, but due to space constraints, these will not be elaborated further.

#### 5.3.2. Quality Security

As depicted in Figure 4b, China’s quality security level demonstrates an upward trend, albeit the overall level remains not high. In contrast, the United States’ food quality security level reveals a relatively flat trend but surpasses that of China. Looking at the internal indicators, all the indicators of food quality security in China have moved in a positive direction during the period from 2000 to 2022. 

Among these, the per capita protein supply and per capita fat supply in China have increased by an average of 27.90% and 38.30%, respectively, and the diversity of the diet has also significantly improved, expected to exceed 50% [73]. Viewed from the angle of nutritional supply, the dietary habits of the Chinese populace are undergoing a swift evolution from a predominantly grain-based diet to a more diversified one, a transformation that has seen the grain supply progressively transition from a sheer quantitative deficit to a structural shortfall, and from an outright caloric inadequacy to a nutritional imbalance [74]. The conventional paradigm of grain production, characterized by a singular pursuit of high yield and increased output, is no longer sufficient to guarantee the effective provision of grain in contemporary China [64]. Relative to China, the food supply in the United States is more plentiful and varied, yet this does not inherently imply dietary balance and health. As per the Dietary Guidelines for Americans, the typical dietary intake of an American is marked by an overconsumption of salt, saturated fats, and sugars [75]. The propensity towards foods with high caloric content but low nutritional value amplifies the susceptibility to chronic diseases. According to data from the U.S. Centers for Disease Control and Prevention (CDC), the 2022 population data shows that in 22 states, the adult obesity prevalence has reached or exceeded 35%. Obesity increases the risk of many other serious health conditions, including heart disease, stroke, type 2 diabetes, certain cancers, and COVID-19 [76]. Hence, the effective balancing of dietary structure and the eradication of nutritional issues pertaining to food security represent the pressing challenges that both China and the United States are presently grappling with.

#### 5.3.3. Circulation Security

**Figure 6:**
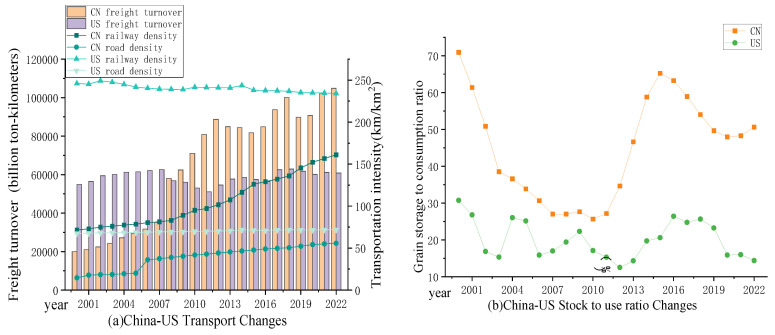
Comparison of grain logistics in China and the United States from 2000 to 2022. Data source: Figure (**a**) the Ministry of Transport of China and the Department of Transportation of the U.S. Figure (**b**) the USDA FSA PSD Database, Breck Agri-Economic Database, ‘*China Rural Statistical Yearbook*’, ‘*China Grain and Materials Reserve Yearbook*’, and ‘*China Statistical Yearbook*’.

The aggregate level of food circulation security in China is witnessing a swift surge, progressively aligning with that of the United States, as demonstrated in Figure 4c. During this period, the level of circulation security in China increased from 0.0625 to 0.1130, an increase of 80.80%, narrowing the gap with the United States, as depicted in Figure 6. 

China is continually hastening the development of its foundational infrastructure for grain logistics, with a steady expansion in the operational distance of its railways and highways, and a gradual escalation in the quantity of goods transported. China has successively constructed eight grain transportation lines, known as the ‘Two Horizontal and Six Vertical’, and leveraging the opportunity of the ‘Belt and Road’ initiative, China has established effective grain cooperation relationships with neighboring countries in Central Asia and Southeast Asia [77]. This has led to the preliminary formation of a modern food logistics network based on ‘Road + Rail + Water Multimodal Transport’, laying a solid foundation for food transportation. Despite China’s freight transportation turnover surpassing that of the United States, under the constraints of limited transportation capacity, grain transportation does not take precedence in railway and highway freight transport. In fact, China continues to grapple with issues in grain transportation, including suboptimal efficiency in grain logistics transport and the lagging standardization of infrastructure construction [78]. In terms of food trade, China has achieved self-sufficiency in its three major grains (rice, corn, and wheat). However, the scale of soybean imports in China is continuously expanding, with the self-sufficiency rate of soybeans persistently declining to 16% [79]. Due to the low yield per unit area of soybeans in China and the slow growth rate, coupled with the instability of the planting area, China will still need to import a large amount in the future to temporarily alleviate the mismatch between supply and demand structures [80]. In contrast, since 2006, genetically modified soybeans have been widely cultivated in American soybean farms. With the promotion of genetically engineered soybeans and precision agriculture technologies, the production of soybeans in the US has achieved rapid growth [81]. Grain reserves can stabilize market supply and demand, thereby enhancing the capacity for grain circulation. In terms of grain reserves, China, compared to the U.S., has exhibited significant fluctuations in the stock–use ratio. Furthermore, the gap between China and the U.S. has been continuously widening since 2012, with China’s stock–use ratio far exceeding that of the U.S., as illustrated in Figure 6b. This indicates that in the event of sudden incidents leading to interruptions in food production supply, China’s emergency supply capabilities are stronger than those of the United States. However, excessive grain production and overstocking can also lead to increased financial subsidies from the state and increased production costs, impact market supply and demand relationships, and pose hidden risks to food security.

#### 5.3.4. Economic Security

As shown in Figure 4d, China’s economic security level has been steadily increasing, while that in the US remains high, with a significant gap still existing between the two. During the period, the level of economic security in China increased by 308.38%, while that in the US rose by 26.98%. 

Since its accession to the WTO in 2001, China has achieved remarkable economic growth through market-oriented system reforms, technological self-reliance, and the implementation of the ‘Belt and Road’ initiative for foreign trade policies, leading to significant changes in both the level and structure of household consumption. Its per capita disposable income has significantly increased, Engel’s coefficient of residents has continuously declined, the productivity per labor unit has noticeably improved, and the volatility of food prices has gradually stabilized. The economic security has dramatically strengthened. Despite the rapid development of China’s agricultural economy, its labor productivity per unit (calculated by the agricultural added value per agricultural worker) remains somewhat weak, markedly trailing behind developed countries such as the US [82]. At present, the surplus degree of agricultural labor, the level of agricultural product processing, and the structure of the agricultural industry are pivotal factors constraining the enhancement of China’s agricultural labor productivity [83]. The focus of China’s agricultural production should transition from intensification, centralization, and scaling towards market-oriented and diversified strategies. In the fight against hunger and poverty, China has contributed to over 70% of the world’s poverty reduction efforts [84]. Throughout a span of over 40 years of poverty reduction strategies, China has undergone four phases: relief-based poverty alleviation (1978–1985); development-guided poverty alleviation (1986–2006); development-focused poverty alleviation and social security systems (2007–2012); and precision poverty alleviation (2013–2020) [85]. In comparison to China, the United States, while having a lower poverty rate under identical standards, leans towards ‘conditional’ poverty alleviation, i.e., for families with children, employment, or those nearing the poverty line. Policies to reduce poverty in the U.S., post the mid-1990s, have intensified inequality amongst the impoverished [86]. Additionally, the U.S. official poverty standard remains a ‘unidimensional indicator’, primarily focused on income [87]. Conversely, China’s poverty alleviation standard is a ‘multidimensional indicator’, thoroughly considering aspects such as sustenance, housing, education, and healthcare, thereby fulfilling survival and basic living necessities while also embodying developmental demands [88]. A decrease in poverty rates and an elevation in the living standards of those with low income can bolster their capacity to access food, which in turn aids in upholding national food security.

#### 5.3.5. Ecological Resource Security

As illustrated in Figure 4e, the level of ecological resource security in China exhibits a slow upward trend. However, due to the excessive use of pesticides and fertilizers, as well as the scarcity of arable land and water resources, the overall improvement is limited. In recent years, the ecological resource security in the US has shown a fluctuating downward trend due to frequent drought disasters and a decrease in arable land resources, but overall, the US surpasses China. 

**Figure 7:**
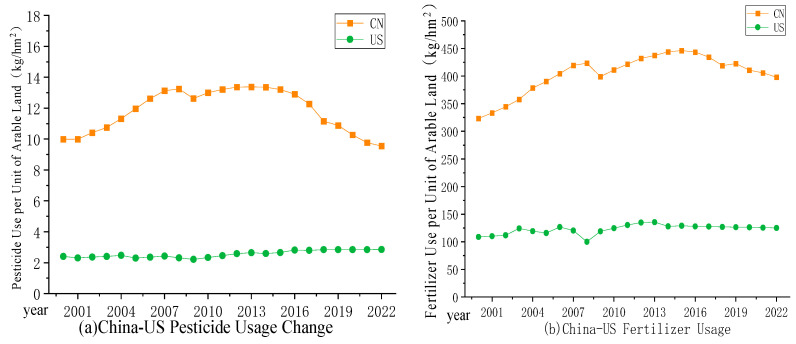
China–US pesticide and fertilizer usage change. Data source: World Bank, FAO, and the National Bureau of Statistics of China.

In the last two decades, the per capita arable land in both China and the United States has been in a state of decline, with decreases of 0.0392 ${hm}^{2}$ and 0.0172 ${hm}^{2},$ respectively [89]. Despite the fact that the rate of decline in the per capita arable land area in the US surpasses that of China, the overall quantity remains greater in the US. With respect to the preservation of cultivable land, China still has deficiencies compared to the US. Examples include insufficient coordination between the central and local governments in the protection of cultivable land, the imperfect management of agricultural functional zones, and an incomplete compensation mechanism for cultivable land protection, as well as a lack of adequate protection for the quality of arable land [90]. Besides arable land resources, the issue of water resource scarcity is equally troubling for China. In 2020, the per capita water resource in China stood at 1993 m^3^ accounting for only 23% of that in the US, but the area of arable land in China that requires irrigation reached 69160.52 ${hm}^{2}$, which is 2.9 times as much as that in the US [91]. Furthermore, a spatial incongruity is observed between the sites of grain production and the provision of water resources in China [92]. The six northern regions of China, namely the Songhua River region, Liao River region, Hai River region, Yellow River region, Huai River region, and the Northwestern Rivers region, constitute the core areas of grain production. However, the total water resources in these regions account for only 21% of the total [93]. Historically, China has managed to continuously increase crop yields through the extensive use of fertilizers and pesticides, feeding 21% of the global population with a mere 7% of arable land, albeit at the cost of severe ecological damage [94]. In an effort to effectively control the use of fertilizers and pesticides, China’s Ministry of Agriculture initiated amendments to the maximum residue limits for pesticides in 2012, and implemented the ‘double reduction’ policy in 2015, launching a ‘zero-growth’ action for the use of fertilizers and pesticides. These measures have led to a noticeable decrease in the per unit use of pesticides and fertilizers in China, yet a gap still persists when compared to developed countries such as the US. In 2022, the per unit use of pesticides and fertilizers in China was 9.54 $kg/{hm}^{2}$ and 398.05 $kg/{hm}^{2}$, respectively, which is 3.34 times and 3.18 times that of the US, as demonstrated in Figure 7. Consequently, enhancing the efficiency of pesticide and fertilizer use, and perfecting the application system of pesticides and fertilizers that balances environmental protection with agricultural productivity are crucial measures for China’s development of sustainable agriculture.

#### 5.3.6. Policy Security

As depicted in Figure 4f, over the past two decades, China has seen a significant enhancement in its policy security level, whereas the policy security level in the US has experienced minimal fluctuations, yet it still maintains a certain disparity with China. 

Analyzing from the perspective of the policy security subsystem, the regulatory quality, government effectiveness, and political stability in China have all exhibited a steady ascending trend, with the speed of growth in governmental efficiency being particularly rapid. The growth of these three indicators, to a certain extent, signifies the enhancement of the Chinese government’s policy implementation capabilities, playing a crucial supporting role in the macro-control of food production. With respect to funding for agricultural research, there has been a marked increase in China over the last two decades. When taking into account the effects of inflation and viewing from the standpoint of purchasing power parity, the total investment in agricultural research in China has now exceeded that of the US, making it the largest agricultural research and development country in the world [95]. Following 2003, China’s No. 1 Central Document has repeatedly underscored the significance of agricultural technology and modernization with the intent to stimulate agricultural innovation, escalate national investment in agricultural R&D, and expedite the edification of modern agriculture [96]. Nonetheless, there is room for improvement in China’s capacity for innovation in agricultural research. The intensity of food and agricultural research and development in China still significantly lags behind the United States, with China’s R&D expenditure not having substantively strengthened [97]. Furthermore, the scientific literacy of Chinese farmers is relatively low, which poses significant challenges to the dissemination of scientific research and technological achievements. According to the data from the third National Agricultural Census of China, as many as 91.7% of agricultural producers and operators have an education level of junior high school or below. The scientific literacy of agricultural technicians in grassroots agricultural technology departments is uneven, and there is a scarcity of high-quality talents, which further reduces the efficiency of promoting scientific research and technological achievements [64]. There is a substantial absolute disparity in agricultural scientific and technological innovation capabilities among different provinces in China, with a serious polarization [98]. The traditional extensive grain production model, which has previously relied on inputs such as pesticides and fertilizers, is no longer sustainable. On the path to modernizing grain production in China, technological innovation should be regarded as the leading force to ensure food security.

### 5.4. Evolution of Obstacles in Food Security

**Figure 8:**
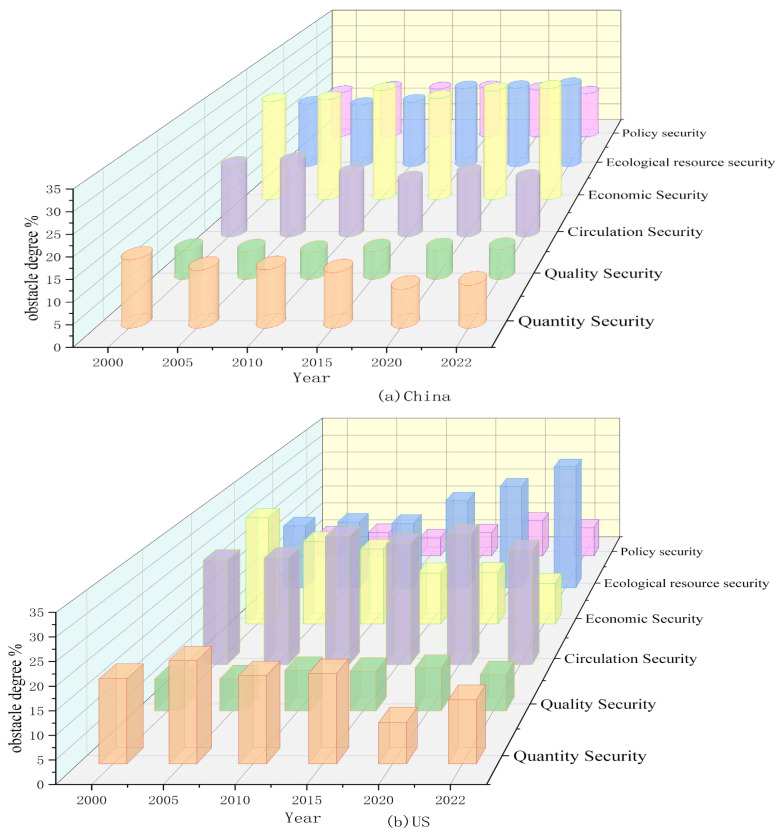
(**a**) Trends in the degree of obstacles in the various dimensions of food security in China. (**b**) Trends in the degree of obstacles in the various dimensions of food security in the United States.

**Figure 9:**
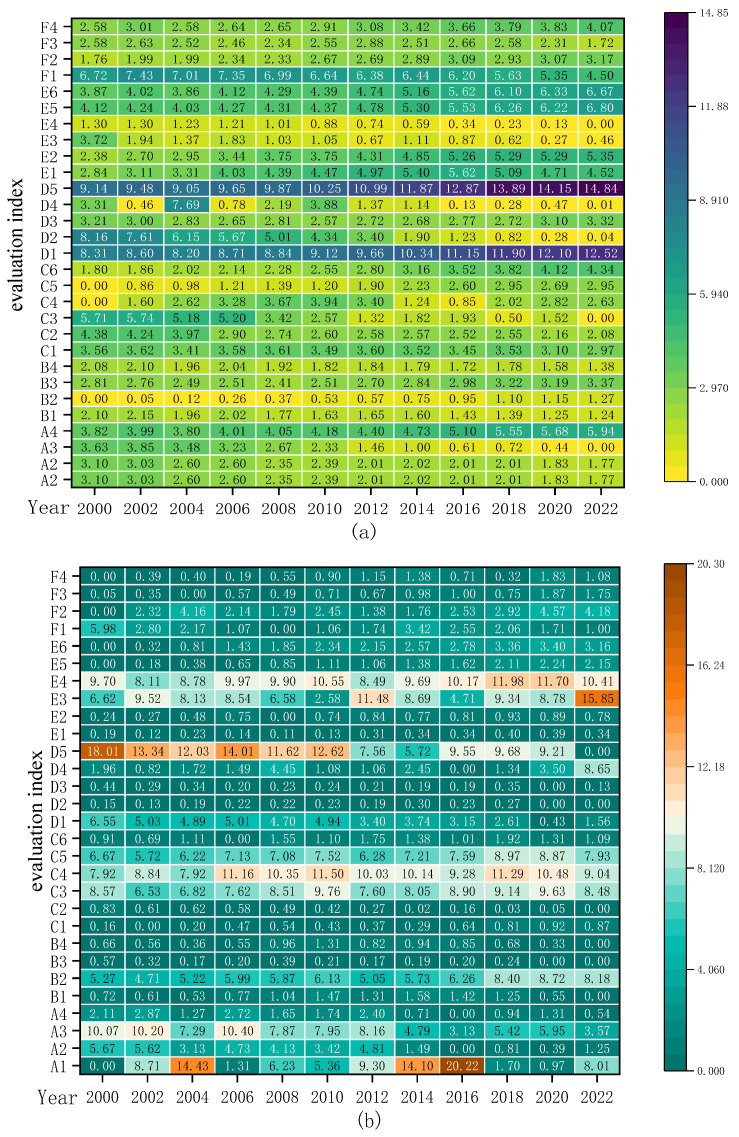
(**a**) Trends in the degree of obstacles for the various indicators of food security in China. (**b**) Trends in the degree of obstacles for the various indicators of food security in the United States.

This section embarks on the six dimensions of food security, employing the obstacle degree model to analyze the hindering factors that constrain the enhancement of the food security levels in China and the United States. The trend in the obstacle degrees in the various subsystems of food security in China and the United States from 2000 to 2020 is illustrated in Figure 8 and Figure 9.

From Figure 8a and Figure 9a, it can be observed that the main obstacles affecting food security in China are concentrated in the economic security subsystem and the ecological resource security subsystem, with their average obstacle degrees reaching 29.17% and 20.61%, respectively, and showing a slow upward trend. Subsequently, the magnitude of the average obstacle degree is in the sequence of circulation security, policy security, quantity security, and quality security. Notably, the obstacle degrees of quantity security and circulation security in China exhibit a gradual decrement trend, descending from 13.55% and 15.45% in 2000, respectively, to 9.77% and 14.96% in 2022. The obstacle degrees of quality security and policy security in China demonstrate a slight oscillatory increase, with the overall change being relatively insignificant. 

With the advancement of agricultural mechanization in China, the widespread adoption of novel planting and breeding technologies, increased financial investments in agriculture, and the expansion of grain sowing areas, the continuous enhancement of grain productivity has led to an unprecedented 19-year consecutive increase in grain production. Consequently, the impact of quantity security on food security has progressively diminished. However, with the transformation of people’s consumption concepts, the pursuit has shifted towards diverse, nutritious, green, and healthy food, no longer merely satisfying the most basic needs for sustenance. The contradiction between the people’s demand for high-quality food and the insufficient total supply, coupled with an unbalanced structure, has become prominent, thereby amplifying the impact on food quality security. In recent years, China’s infrastructure for food transportation and storage has been continuously improved, and the logistic routes for food transportation have been constantly expanded, leading to a decrease in the constraints of food circulation on China’s food security. However, there have been issues due to the current uneven efficiency of grain transportation across the country, limited cross-regional grain transportation capacity, and risks to the import of high-demand foods such as soybeans due to the recent China–US trade wars and the COVID-19 pandemic. Thus, the potential for reducing the impact of circulation security on food security is limited. Economic security and ecological resource security are the two major factors currently affecting China’s food security. On one hand, in recent years, with the implementation of a series of supportive policies such as rural revitalization and targeted poverty alleviation, China has eradicated absolute poverty. However, China’s poverty alleviation work still faces significant challenges, such as multidimensional relative poverty in urban and rural areas, and a lack of intrinsic motivation in impoverished areas [99]. Additionally, despite an overall increase in per capita income and labor productivity, the profitability of grain farming has been growing at a sluggish pace due to the rising costs of grain cultivation and declining grain prices, leading to a severe phenomenon of grain farmers’ attrition. On the other hand, although the Chinese government has implemented a ‘trinity protection system’ for the quantity, quality, and ecology of cultivated land, as well as measures such as the ‘South-to-North Water Diversion’ project to alleviate the reduction in arable land and the uneven distribution of water resources, it has been unable to resolve the contradictions in agricultural production. Furthermore, the ‘Double Reduction’ policy issued by the Ministry of Agriculture in 2015 has effectively reduced the use of chemical fertilizers and pesticides. However, due to the low productivity of the land, ordinary farmers still resort to using chemical fertilizers and pesticides to increase yield. This has caused severe damage to China’s ecological environment. China’s food security is under threat from unsustainable practices.

From Figure 8b and Figure 9b, the main obstacles affecting U.S. food security are concentrated in the circulation security subsystem and the ecological resource subsystem, with average obstacle degrees reaching 27.44% and 22.01%, respectively. Moreover, the obstacle degree of ecological security is continuously increasing, rising from 16.74% in 2000 to 32.69%. The remaining obstacles, in order, are quantity security, economic security, quality security, and policy security. Subsequently, the magnitude of the average obstacle degree is in the sequence of quantity security, economic security, quality security, and policy security. Among them, the obstacle degrees of economic security and quantity security are continuously decreasing, falling from 27.11% and 17.83% in 2000 to 10.33% and 13.36% in 2022, respectively. Meanwhile, the obstacle degrees of quality security and policy security show a slight upward trend, rising to 8.18% and 8.01%, respectively, in 2022. 

Similar to China, the impact of ecological resource security on food security in the United States is gradually expanding, and the influence of circulation security on food security remains high. Unlike China, the impact of economic security on food security in the United States is gradually diminishing. In recent years, climate change has posed a pervasive and increasingly severe threat to the biodiversity and ecosystems of the US. Rising temperatures and alterations in precipitation patterns, along with extreme drought, are transforming the structure and function of U.S. ecosystems, with implications for crop yields [100]. Simultaneously, unsustainable irrigation practices are progressively impacting the enduring utilization of stream water resources in the western United States [101]. The influence of ecological resource security on the US’ food security is gradually intensifying. As the world’s largest exporter of grains, the United States relies heavily on globalized food supply chains, with a significant portion of its produce earmarked for international trade. However, the combined effects of the global pandemic, increasing pressure from the climate crisis, soaring energy and fertilizer prices, and escalating conflicts have disrupted the supply chain. This has gradually increased the impact of circulation security on the United States. Due to the role of federal income support and nutritional assistance programs, the impact of economic security on U.S. food security may be gradually decreasing. These programs help increase food security rates by providing additional income or food benefits to low-income families. For instance, during the COVID-19 pandemic in 2019, stimulus spending and the expansion of SNAP benefits helped reduce the rate of household food insufficiency in 2020 and 2018 [102].

## 6. Discussion and Conclusions

In this study, we expanded the connotation of food security, constructed a more comprehensive and systematic food security assessment framework, analyzed the development of food security in China and the United States since 2000 from a systems science perspective, and elucidated the changes and existing disparities in food security between the two countries in conjunction with national policies. Initially, this paper establishes a comprehensive framework for assessing food security, encompassing six dimensions: quantity security, quality security, circulation security, economic security, ecological resource security, and policy security. The CRITIC–MEREC–MARCOS model is subsequently employed to holistically evaluate the developmental status of food security in both China and the United States from 2000 to 2022. Building upon this foundation, the obstacle degree model is utilized to identify key impediments influencing food security in China and the US. The research findings indicate that China’s level of food security during this period exhibited a slight fluctuation initially, followed by a gradual and steady upward trend, demonstrating an overall positive trajectory with a diminishing gap compared to the United States. However, when scrutinizing each dimension, China still exhibits pronounced disadvantages in economic security, ecological resource security, and policy security compared to the United States. Concurrently, due to limited agricultural labor productivity, scarce water and soil resources, and inefficiencies in the use of fertilizers and pesticides, China’s food security is constrained by economic and environmental factors. The constraints of economic security and ecological resource security on China’s food security show an increasing trend year by year. In contrast, for the United States, the impact of disruptions in the international trade for grains, coupled with an escalating frequency of drought disasters, is increasingly highlighting the significance of circulation security and ecological resource security in influencing food security.

In the results of this study, although both China and the United States face challenges in food security, the issues exposed in China are more pronounced. The food security challenges confronted by China are akin to those experienced by many rapidly developing nations. Firstly, there is an inadequacy in the coordinated development of food production and environmental resource preservation. Despite being a pivotal global player in food production, China has made significant strides in grain output often accompanied by the depletion of water and soil resources and the degradation of ecosystems [103]. Amidst the constraints of limited agricultural productivity, the continuous growth in China’s grain production is sustained by practices such as excessive land utilization, the over-extraction of water resources, the disproportionate use of fertilizers and pesticides, and intensive labor practices. Secondly, there exists an imbalance between the structure of food supply and the residents’ consumption patterns. On the supply side, China currently faces an anomaly characterized by the ‘three highs’—high production, high stockpiles, and high imports. Surpluses in wheat, rice, and corn contrast sharply with a severe shortage in soybean production, with soybean supply heavily reliant on imports from countries such as Brazil, the United States, and Argentina. On the demand side, there is a shift from a singular emphasis on staple grain requirements to diverse needs such as meat, eggs, dairy, and aquatic products. This transition extends from fulfilling basic nutritional needs to an emphasis on health, leading consumers to seek functional food products rich in selenium, zinc, and other elements associated with lipid and blood sugar reduction. Moreover, there is a shift from material satisfaction to the pursuit of higher-level spiritual needs, as consumers transition from meeting their fundamental dietary requirements to a demand for pet rearing.

Looking ahead, with the globalization of food trade, the transmissibility, interconnectivity, and transnational nature of food security issues will become increasingly prominent. In recent years, the resurgence of global trade protectionism, unilateralism, and hegemonism, coupled with frequent major geopolitical events and emergencies such as the COVID-19 pandemic and Russo-Ukrainian conflicts, have dealt a severe blow to global agricultural economic and trade cooperation. As agricultural powerhouses and major agricultural trade nations, China and the United States hold a pivotal position in global agricultural trade. However, the economic and trade frictions between China and the United States, whether in the imposition of tariffs on agricultural product imports or in the signing and implementation of the first phase of the trade agreement, have continuously altered the global agricultural trade landscape, exacerbating inequalities in the pursuit of sustainable development goals worldwide [104]. As competition intensifies, the current CN–US rivalry has transcended economic conflict, evolving towards a more comprehensive confrontation, expanding from trade disputes to battles over cutting-edge technology, regional strategies, and divergent value systems [105]. Although there remain divergences between China and the United States in areas such as geopolitics and economic systems, there still exists a space for cooperation between the two.

The agriculture of both nations exhibits strong complementarity. The United States, with its cost advantages in producing agricultural products such as soybeans, corn, and cotton, can meet China’s demand for certain scarce agricultural products. China, primarily exporting labor-intensive agricultural products, can also satisfy the United States’ demand for distinctive agricultural products such as citrus. Furthermore, in addressing climate issues closely related to food security, China and the United States have successively announced their goals and timelines for ‘carbon peaking’ and ‘carbon neutrality’. However, the realization of these goals is inseparable from the development of low-carbon technologies. On this basis, China and the United States share common interests and have a solid foundation for cooperation and prospects for deepening cooperation in areas such as the energy industry and green economy. As two countries with significant influence in the world, consensus on climate issues between China and the United States can drive the actions of countries worldwide, injecting powerful momentum into global climate governance. In the current situation where food crises, energy crises, and geopolitical challenges coexist, China and the United States should fulfill their responsibilities as major powers, seek common ground while reserving differences, cooperate with countries worldwide, and respond to global challenges.

This study still has several limitations. Firstly, due to data constraints, this research could not conduct a detailed investigation into the regional spatial distribution differences in food security levels between the two countries. Both China and the United States are vast nations with significant regional differences in natural and social conditions, leading to varying capacities for food production, transportation, and consumption across different areas. Changes in national overall food security may not fully reflect the balanced development of food security levels in each region. Therefore, future research should consider both the national and regional perspectives on food security. Secondly, this study did not adequately consider the impact of food trade on food security. The globalization of food trade has tightly interconnected the food supply chains of various countries. A country’s food security is influenced not only by its own food production and consumption but also by the food production, export, and import policies of its trading partners. Thus, future research could analyze the complexity and diversity of food trade by examining the types of traded commodities, source countries, and trade routes to gain a more comprehensive understanding of their impact on food security.

## Figures and Tables

**Table 1:** Food security evaluation indicator system.

First-Level Indicators	Second-Level Indicators/Unit	Index Definition/Explanation
Quantity Security (A)	A1: Grain production volatility (%)	(Grain yield—Average grain yield in the last 5 years)/Average grain yield in the last 5 years
	A2: Grain yield per unit area (kg/hm^2^)	Total grain production/total grain sown area
	A3: Total food production (t)	Total production of grains, beans, and tubers
	A4: Per capita grain possession (kg/person)	Total grain production/total population
Quality Security (B)	B1: Per capita protein supply (g/day/person)	Total food protein supply/(total population × 365)
	B2: Per capita fat supply (g/day/person)	Total food fat supply/(total population × 365)
	B3: Dietary diversity (%)	Share of dietary energy supply derived from cereals, roots, and tubers
	B4: Dietary energy supply adequacy (%)	Dietary energy supply/dietary energy requirement
Circulation Security (C)	C1: Density of railway routes (km/km^2^)	Railway operating mileage/total land area
	C2: Density of highway routes (km/km^2^)	Highway operating mileage/total land area
	C3: Freight turnover (tons-km)	Total freight tonnage × freight average haul distance
	C4: Stock to use ratio (%)	End-of-year grain stockpile/next year’s grain consumption
	C5: Per capita grain consumption (kg)	Total grain consumption/total population
	C6: Grain self-sufficiency rate (%)	Total grain production/(total grain production + imports-exports)
Economic Security (D)	D1: Per capita disposable income (USD)	Reflecting the actual consumption level of residents
	D2: Poverty incidence rate (%)	Number of people below the poverty line/total population
	D3: Engel’s coefficient for residents (%)	Food consumption expenditure/total consumption expenditure
	D4: Food price volatility (%)	The consumer price index for food reflects market price fluctuations
	D5: Agricultural labor productivity (USD/person)	Total agricultural production value/number of agricultural workers
Ecological Resource Security (E)	E1: Pesticide use per unit of arable land (kg/hm^2^)	Pesticide consumption/total arable land
	E2: Fertilizer use per unit of arable land (kg/hm^2^)	Consumption of chemical fertilizer/total arable Land
	E3: Proportion of drought-affected area (%)	Proportion of agricultural crops affected by drought area
	E4: Effective irrigation area (hm^2^)	Area of arable land with regular irrigation for agricultural production
	E5: Per capita water resources (m^3^/person)	Total freshwater resources/total population
	E6: Arable land per capita (hm^2^/person)	Total arable land area/total population
Policy Security (F)	F1: Agricultural research funding expenditure (USD)	Reflecting the government’s investment intensity in agricultural research
	F2: Political stability	Measuring the degree of political stability of a country
	F3: Government effectiveness	Measuring efficiency in policy formulation and implementation
	F4: Regulatory quality	Measuring the regulatory strength of policy formulation and implementation

**Table 2:** Data sources.

Country	Indicator	Data Sources
China	A1, A2, A3, A4, C5, D1, D3, D4, D5, E1, E2, E3, E4, E5, and E6	the National Bureau of Statistics and calculated by the authors
	B1, B2, B3, and B4	FAO
	C1, C2, and C3	the National Bureau of Statistics, the Ministry of Transport of China, and calculated by the authors
	C4 and C6	*the China Rural Statistical Yearbook, the China Grain and Materials Reserve Yearbook, the China Statistical Yearbook*, and Bric Agricultural Data Intelligence Terminal and calculated by the authors
	D2, F2, F3, and F4	the World Bank
	F1	*the China Statistical Yearbook On Science and Technology*
US	A1, A2, A3, A4, C4, C5, C6, E4, and F1	the U.S. Department of Agriculture, *Agricultural Statistics, Acreage* and calculated by the authors
	B1, B2, B3, B4, E1, and E5	FAO
	C1, C2, and C3	the U.S. Department of Transportation and calculated by the authors
	D1, D3, D4, and D5	the U.S. Bureau of Economic Analysis, the U.S. Department of Labor and calculated by the authors
	D2, E2, E6, F2, F3, and F4	the World Bank
	E3	the U.S. National Integrated Drought Information System and calculated by the authors

**Table 3:** Weight of food security indicators.

First-Level Indicator	Second-Level Indicators	CRITIC Weighting	MEREC Weighting	Combination Weighting
Quantity Security (A) 14.82%	A1	7.07%	4.36%	5.72%
	A2	2.01%	2.79%	2.40%
	A3	3.22%	4.16%	3.69%
	A4	3.16%	2.87%	3.02%
Quality Security (B) 7.63%	B1	1.57%	1.71%	1.64%
	B2	2.67%	1.75%	2.21%
	B3	2.00%	2.34%	2.17%
	B4	1.65%	1.56%	1.61%
Circulation Security (C) 18.20%	C1	2.99%	2.51%	2.75%
	C2	2.57%	4.20%	3.39%
	C3	5.13%	3.70%	4.41%
	C4	3.56%	3.33%	3.45%
	C5	2.30%	2.03%	2.16%
	C6	2.14%	1.94%	2.04%
Economic Security (D) 28.26%	D1	5.12%	7.72%	6.42%
	D2	5.40%	7.21%	6.31%
	D3	2.71%	2.25%	2.48%
	D4	5.20%	6.78%	5.99%
	D5	5.86%	8.28%	7.07%
Ecological Resource Security (E) 19.48%	E1	3.51%	2.81%	3.16%
	E2	3.52%	2.18%	2.85%
	E3	4.58%	3.97%	4.28%
	E4	3.66%	2.22%	2.94%
	E5	3.46%	2.92%	3.19%
	E6	3.32%	2.80%	3.06%
Policy Security (F) 11.61%	F1	4.83%	6.25%	5.54%
	F2	2.77%	0.89%	1.83%
	F3	2.91%	1.09%	2.00%
	F4	3.11%	1.38%	2.25%

## Data Availability

The raw data supporting the conclusions of this article will be made available by the authors on request.
